# Surgical Management of a Prominent Adduction-Induced Upshoot in Duane Retraction Syndrome Type III: A Case Report

**DOI:** 10.7759/cureus.87397

**Published:** 2025-07-06

**Authors:** Miwa Komori, Miho Sato, Akari Arakawa, Hiroki Kaneko, Akiko Hikoya

**Affiliations:** 1 Department of Ophthalmology, Hamamatsu University School of Medicine, Hamamatsu, JPN

**Keywords:** abnormal ocular movement, cosmetic concern, duane retraction syndrome, forced duction test, lateral rectus muscle recession, spring-back balance test, strabismus surgery, superior rectus muscle recession, upshoot, vertical deviation

## Abstract

Duane retraction syndrome (DRS) with good primary gaze alignment and no abnormal head posture is often managed conservatively to avoid worsening alignment. However, surgical intervention may be considered when significant cosmetic concerns are present. We present the case of a 29-year-old male patient with type III DRS of the right eye and a prominent upshoot. Despite having good primary gaze alignment, the patient desired cosmetic improvement. Forced duction testing revealed restrictions in multiple directions. Based on the spring-back balance test, we performed an 8.0 mm recession of the lateral rectus muscle and a 6.0 mm recession with partial nasal transposition of the superior rectus muscle. One year postoperatively, the vertical deviation had improved to 8 prism diopters (PD) at distance and 12 PD at near. The upshoot was significantly reduced, and there were no new limitations or diplopia. The patient achieved cosmetic satisfaction. Combined lateral and superior rectus recession effectively reduced an adduction-induced upshoot in DRS type III. Surgery may be considered for patients with good primary gaze alignment and significant cosmetic concerns.

## Introduction

Duane retraction syndrome (DRS) is a congenital disorder of eye movements caused by an absent or underdeveloped abducens nerve. It was first described by Duane in 1905 [1]. DRS is characterized by limited abduction alone or a limitation of both abduction and adduction combined. Abnormal nerve signals from the oculomotor nerve to the lateral rectus muscle often cause globe retraction, narrowing of the palpebral fissure, and vertical globe deviations, such as upshoots and downshoots, when adduction occurs. Additionally, developmental abnormalities of the extraocular muscles may result in fibrosis and contracture [2]. DRS has recently been categorized as one of the congenital cranial dysinnervation disorders (CCDDs). Magnetic resonance imaging (MRI) allows for evaluation of the presence of the abducens nerve [3].

DRS is classified into three types based on the pattern of motility limitation: type I with abduction limitation, type II with adduction limitation, and type III with limitation of both. In clinical practice, treatment is determined based on eye position in primary gaze and the presence or absence of compensatory head posture. Medial rectus recession is often performed for esotropia and lateral rectus muscle recession for exotropia [4-6]. If the eye position in primary gaze is good and there is no abnormal head posture, observation is often chosen to avoid worsening the alignment in primary gaze. However, when significant vertical deviation causes cosmetic concern, surgical intervention may be considered [7].

Here, we present a case of DRS type III with no abnormal head posture, only mild vertical deviation in primary gaze, and a prominent upshoot on adduction. The patient desired cosmetic improvement and underwent lateral and superior rectus muscle recession, resulting in a favorable outcome.

## Case presentation

**Figure 1:**
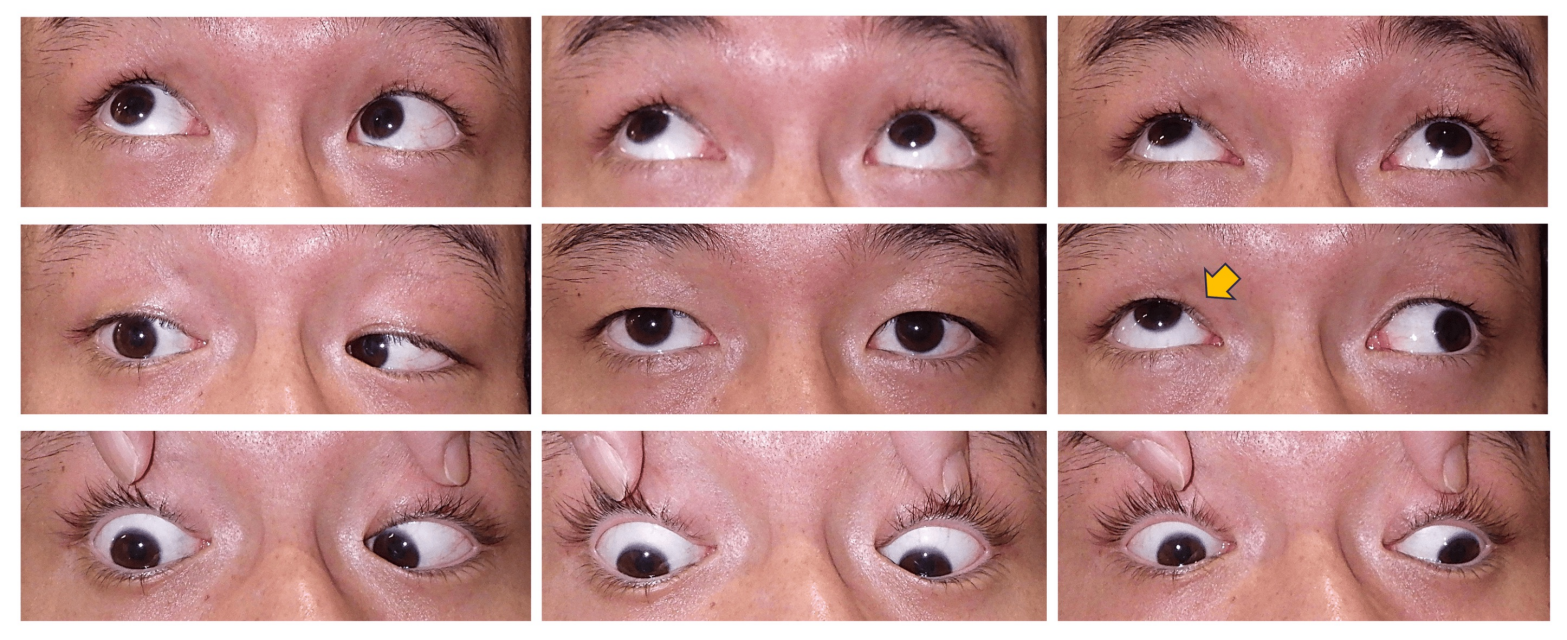
Preoperative nine-gaze photographs Mild hypertropia in the primary gaze position and a prominent upshoot during left gaze (yellow arrow).

**Figure 2:**
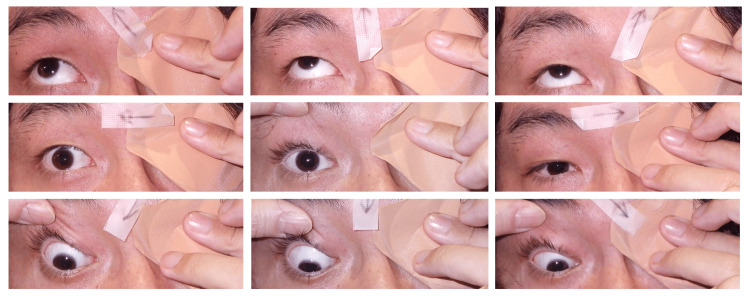
Preoperative duction photographs of the right eye Limitation of abduction and adduction (-2 each), with narrowing of the palpebral fissure on left gaze.

A 29-year-old male presented with an abnormal position of his right eye that was particularly noticeable during a left gaze. He had been aware of the condition since childhood. He had been diagnosed with DRS during high school and was managed conservatively due to good alignment in primary gaze and the anticipated difficulty of achieving complete correction. Recently, he sought cosmetic improvement and was referred to our clinic. Upon examination, his best-corrected visual acuity was 20/20 in both eyes. The alternate prism cover test (APCT) revealed 6 prism diopters (PD) of exotropia and 18 PD of right hypertropia at a distance; 14 PD of intermittent exotropia and 16 PD of right hypertropia at near with left eye fixation. The Stereo Fly Test demonstrated fly (+), animals 2/3, and circles 0/9. Cyclophorometry revealed 4° of extorsion. There was no diplopia in primary gaze. An ocular motility examination revealed a -2 limitation of both adduction and abduction in the right eye, with a prominent upshoot and narrowing of the palpebral fissure during left gaze (Figure 1 and Figure 2).

These findings led to a diagnosis of DRS type III in the right eye. Although the deviation in primary gaze was mild, the patient's desire for cosmetic improvement led to the decision to perform surgery under general anesthesia. Initially, a combined recession of the lateral and medial rectus muscles was considered. However, the patient was informed preoperatively that the final surgical procedure would be determined intraoperatively based on forced duction and spring-back balance test results.

Intraoperative forced duction testing revealed no limitations in the left eye but significant restrictions in adduction and abduction in the right eye. Elevation was possible, but depression was also restricted. These findings suggested contracture of the right medial, lateral, and superior rectus muscles. We proceeded with recession of the lateral and superior rectus muscles. During surgery, the lateral and superior rectus muscles were disinserted from their insertion sites. Repeat forced duction testing revealed that the adduction and depression restrictions had resolved. The lateral rectus (8.0 mm) and superior rectus (6.0 mm) muscles were recessed on the right eye. One-third of the superior rectus muscle's width was transposed nasally and temporarily secured in place. After confirming good alignment with the spring-back balance test [8], the muscles were permanently sutured.

**Figure 3:**
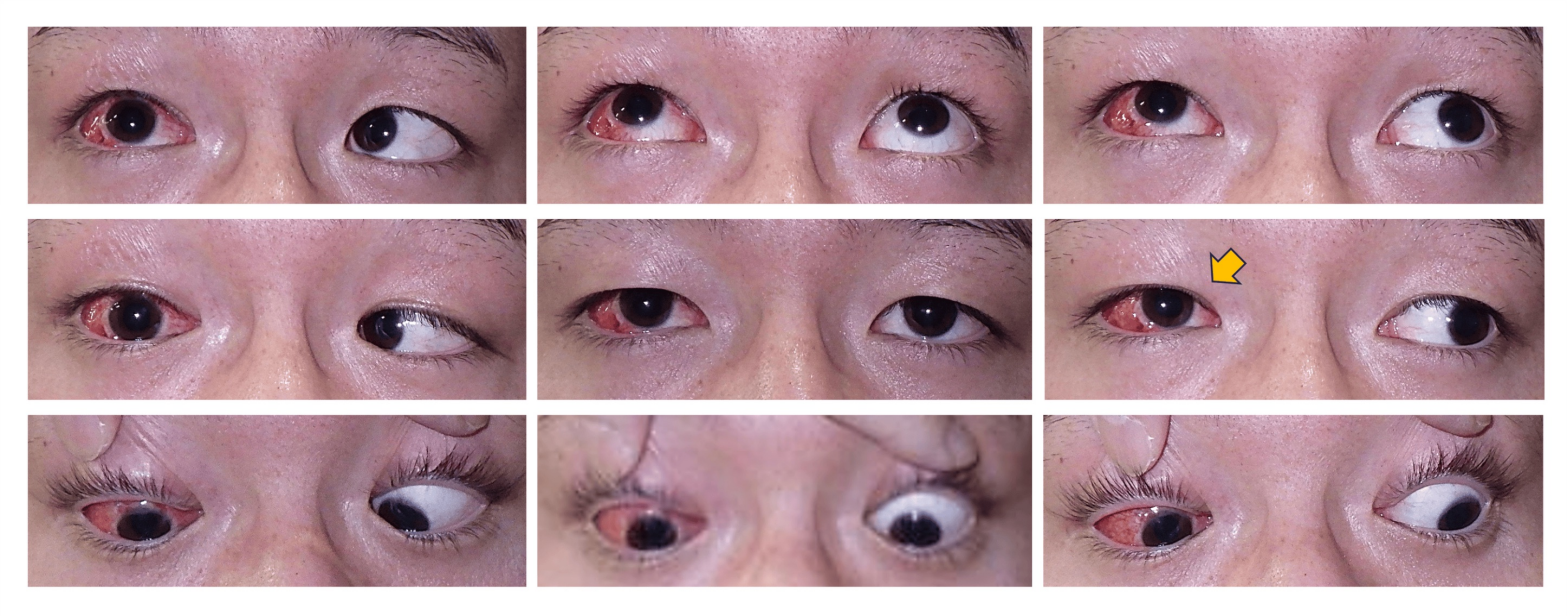
Postoperative nine-gaze photographs at one month Reduced hypertropia and improved upshoot (indicated by yellow arrow).

One week after surgery, APCT showed 5 PD of right hypertropia at a distance and 8 PD at near with left eye fixation. There was a marked improvement in the upshoot in primary position and during left gaze. One month postoperatively, the APCT revealed 3 PD of right hypertropia at a distance and 8 PD at near (with left eye fixation). Torsional deviation was not quantitatively measured postoperatively. Nevertheless, the patient did not report any torsional diplopia or visual discomfort, suggesting that the impact on ocular torsion was minimal. There was no worsening of limitations in abduction or elevation, and the elevation during adduction was clearly reduced (Figure 3).

**Figure 4:**
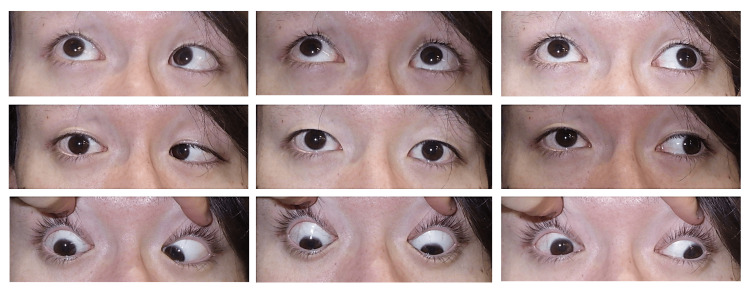
Postoperative nine-gaze photographs at one year Stable alignment and sustained cosmetic improvement.

One year after surgery, APCT showed stable alignment with 8 PD of right hypertropia at a distance and 12 PD at near (with left eye fixation). No diplopia was observed in the primary gaze. The patient was highly satisfied with the cosmetic result (Figure 4).

The Stereo Fly Test remained unchanged from preoperative levels: fly (+), animals 3/3, and circles 0/9. This indicates preserved stereopsis without notable improvement.

## Discussion

Vertical globe deviations in DRS, such as upshoots and downshoots, are attributed to two main mechanisms: mechanical and innervational [4]. Mechanical factors include the "bridle effect," in which a tight lateral rectus muscle slips over the globe during adduction, resulting in abrupt vertical movement. Innervational factors involve the co-contraction of the lateral rectus muscle with either the superior or inferior rectus muscles due to aberrant innervation, as confirmed by electromyography studies [9]. Clinically, two features have been proposed to differentiate between the two mechanisms [4]: 1. Vertical deviation due to mechanical factors appears only during adduction; innervational causes show a gradual increase in vertical deviation from primary gaze to adduction. 2. In primary gaze, mechanical causes usually result in mild vertical deviation, whereas innervational causes are more likely to present with large-angle deviation. However, these mechanisms often coexist, and an electromyographic evaluation is necessary for a definitive diagnosis.

Various surgical approaches have been reported for DRS. These include large lateral rectus muscle recession [4], combined medial and lateral rectus muscle recession [10], lateral rectus muscle Y-splitting with recession [11,12], vertical rectus recession [4,13,14], Nishida's procedure [15], vertical rectus transposition [16,17], periosteal fixation of lateral rectus muscle [17], and anterior-nasal transposition of the inferior oblique muscle [18]. Kraft [4] and Gaur [6] discuss these treatment strategies as practical guidelines, but a standardized surgical procedure has not yet been established. Among these techniques, Y-splitting of the lateral rectus muscle is considered effective in cases dominated by mechanical factors [11,12]. This technique involves splitting the muscle approximately 10 mm along its insertion and reattaching the halves superiorly and inferiorly to reduce vertical slippage. However, this method has drawbacks, such as difficulty quantifying the effect and the potential for adhesions to complicate future surgeries. For innervational causes, recession of the superior rectus muscle for upshoot or the inferior rectus muscle for downshoot has been reported to be effective [4,13,14]. These procedures are relatively simple, can be combined with lateral rectus muscle recession, and are suitable for reoperations.

In our case, intraoperative forced duction testing suggested the involvement of mechanical factors due to the contracture of the medial and lateral rectus muscles, as well as the superior rectus muscle. Furthermore, the gradually increasing vertical deviation from primary gaze to adduction and the 18 PD right hypertropia in primary gaze suggested a contribution from innervational factors. Although electromyography was not performed, we decided to perform a lateral and superior rectus muscle recession. To address the exotropia in primary gaze and potentially improve adduction, one-third of the superior rectus muscle was transposed nasally. As a result, both horizontal and vertical deviations in primary gaze improved. Despite observing medial rectus muscle contracture intraoperatively, we avoided surgical intervention on this muscle to reduce the risk of anterior segment ischemia. To determine the appropriate amount of recession for both the lateral and superior rectus muscles, we utilized Jampolsky’s spring-back balance test intraoperatively, which is particularly useful when standard quantification is difficult [8,19].

Although surgery is often avoided in patients with good alignment in primary gaze, it may be warranted when cosmetic concerns or symptomatic incomitant strabismus are significant. Phanphruk et al. reported favorable outcomes in orthophoric patients who presented with such concerns, supporting the efficacy of surgery without compromising the primary position [7]. Our findings support this perspective by demonstrating that surgical intervention can be beneficial even when there is no abnormal head posture or large-angle deviation.

## Conclusions

Recession of the lateral and superior rectus muscles effectively improved a prominent adduction-induced upshoot in a patient with DRS type III. This case highlights the importance of individualized surgical planning based on intraoperative findings. Even in cases with good primary gaze alignment, surgical intervention can be valuable for achieving favorable cosmetic results.
